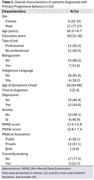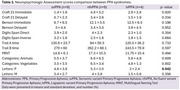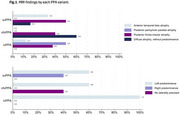# Clinical, Neuropsychological, and Neuroimaging Features of Peruvian Patients with Primary Progressive Aphasia

**DOI:** 10.1002/alz70857_104613

**Published:** 2025-12-24

**Authors:** Diego Bustamante‐Paytan, José Carlos Huilca, Gregory Brown, Maria Fe Albujar‐Pereira, Katherine Aguero, Graciet Verastegui, Zadith Yauri, Pamela Bartolo, Daniela Bendezu, Karol Melissa Lipa‐Pari, Rosa Montesinos, Nilton Custodio

**Affiliations:** ^1^ Unidad de Investigación de Deterioro Cognitivo y Prevención de Demencia, Instituto Peruano de Neurociencias, Lima, Lima, Peru; ^2^ Unidad de Investigación de Deterioro Cognitivo y Prevención de Demencia, Instituto Peruano de Neurociencias, Lima, Peru, Lima, Lima, Peru; ^3^ University of California, San Francisco, San Francisco, CA, USA; ^4^ Instituto Peruano de Neurociencias, Lima, Lima, Peru; ^5^ Unidad de Investigación de Deterioro Cognitivo y Prevención de Demencia, Instituto Peruano de Neurociencias, Lima, Peru; ^6^ Equilibria, Lima, Lima, Peru; ^7^ Hospital Nacional Cayetano Heredia, Lima, Lima, Peru; ^8^ Universidad de San Martín de Porres, Facultad de Medicina, Centro de Investigación del Envejecimiento, Lima, Lima, Peru; ^9^ Unidad de Investigación y Docencia, Equilibria, Lima, Peru; ^10^ Unidad de Investigación y Docencia, Equilibria, Lima, Lima, Peru

## Abstract

**Background:**

Primary progressive aphasias (PPA) are a group of neurodegenerative syndromes primarily characterized by the progressive loss of language. Despite established diagnostic criteria, identification can be challenging, especially in regions with limited resources and few studies, such as Latin America. This study aims to describe the clinical, neuropsychological, and radiological characteristics of a cohort of Peruvian patients diagnosed with PPA.

**Method:**

A retrospective study was conducted on patients diagnosed with PPA at a reference center in Lima, Peru. The 2011 international consensus criteria for PPA were applied. Neuropsychological and clinical assessments, along with magnetic resonance imaging (MRI), were used to confirm the diagnosis. Descriptive statistical analyses were performed for sociodemographic, clinical, and radiological variables. ANOVA was used to assess differences in the neuropsychological battery scores between PPA syndrome groups. IRB approval was obtained for this study

**Result:**

A total of 22 patients were included in the study. The average age of participants was 65.3 (SD 8.7), and 77.3% were male. Semantic and non‐fluent variants were the most frequent PPA subtype with 9 patients respectively. The UDS assessment revealed significant differences were observed in the Letters: P task, where the svPPA and nfvPPA groups performed significantly better than the lvPPA group (*p* = 0.0415). Regarding MRI findings, a high proportion of patients (60%) with nfvPPA presented diffuse atrophy, without predominance.

**Conclusion:**

This study highlights the clinical, neuropsychological, and radiological characteristics of Peruvian patients with PPA, contributing valuable data from a region with limited research in this field. Comprehensive evaluations in resource‐limited settings to improve diagnostic accuracy and understanding of PPA in diverse populations are needed. Further studies are needed for better characterization of these patients in the region.